# Securely stressed: association between attachment and empathic stress in romantic couples

**DOI:** 10.1038/s41598-025-13970-9

**Published:** 2025-09-12

**Authors:** Mathilde Gallistl, Lydia Handke, Melanie Kungl, Sandra Gabler, Ilona Croy, Pascal Vrticka, Veronika Engert

**Affiliations:** 1 Social Stress and Family Health Research Group, Max Planck Institute for Human Cognitive and Brain Sciences, Stephanstr. 1A, 04103 Leipzig, Germany; 2 Department of Clinical Psychology, Friedrich-Schiller-Universität Jena, Jena, Germany; 3 Department of Psychology, Friedrich-Alexander-Universität Erlangen-Nürnberg, Erlangen, Germany; 4 German Center for Mental Health (DZPG), Partner Site Halle-Jena-Magdeburg, Jena, Germany; 5 Department of Psychology, University of Essex, Colchester, CO4 3SQ UK; 6 Institute for Psychosocial Medicine, Psychotherapy and Psychooncology, Jena University Hospital, Friedrich-Schiller-University, Jena, Germany

**Keywords:** Neuroscience, Social neuroscience, Empathy, Psychology, Human behaviour

## Abstract

Stress-related disorders are common in modern societies. What adds to the burden is empathic stress, arising when observing another’s stress elicits a stress response in the observer. In romantic couples, we investigated the association between empathic stress and adult attachment—a deep emotional bond between partners—to understand its inherent facets of risk and resilience. Psychosocial stress was induced in one partner (“target”) while the other passively observed the situation (“observer”). Stress reactivity was measured in both partners via salivary cortisol, heart rate, high-frequency heart rate variability, and questionnaires. Observers’ attachment representations were assessed using the Adult Attachment Interview. We found higher cortisol resonance—that is, proportionality in stress reactivity in targets and observers—in dyads with securely as opposed to insecurely attached (specifically insecure-dismissing) observers. Consistent with attachment as a resilience factor, our results suggest that securely attached individuals are physiologically more in tune with their partners during psychosocially stressful situations, possibly allowing for mutual understanding and triggering supportive behavior. However, suggesting a potential risk inherent to attachment security, in contexts of frequent or extreme partner stress, securely attached individuals may also be prone to excessive empathic stress activation and subsequent health impairments.

## Introduction

Stress is ubiquitous in Western societies. Chronic stress is associated with severe psychological and somatic health risks^1–3^, including depression, anxiety, cardiovascular, metabolic, and autoimmune diseases^4^. One less obvious dimension of stress is empathic stress, where individuals experience stress through others^5^, a phenomenon of profound relevance in our interconnected lives. Attachment, a key component of how we connect with and seek help from others under distress, plays a crucial role as a resilience factor in stress and relationships, particularly in how individuals cope with stress and adversity^6^. In the current study, we examined empathic stress in relation to attachment, focusing on the dual role of attachment as both a resilience and a risk factor within romantic couples.

Stress occurs when an individual perceives a threat to homeostasis (i.e., a state of balance within the body and brain), triggering interdependent physiological and psychological responses^1^. The primary physiological stress systems are the sympathetic-adrenal medullary (SAM) axis, inducing an immediate fight-or-flight response, and the hypothalamic–pituitary–adrenal (HPA) axis, regulating the subsequent slower and longer-lasting release of the main stress hormone cortisol. Humans tend to synchronize emotionally, behaviorally and even on a physiological level with those around them in various situations^7^, including during stress^5,8^. This phenomenon, known as bio-behavioral synchrony (BBS), reflects how individuals’ physiological and behavioral states align with the physiological and behavioral states of others in their environment^9^.

The experience of stress through others is known as empathic stress. Empathic stress is comprised of two components: stress resonance and vicarious stress. Stress resonance involves the synchronized activation of stress response systems across individuals. It has been found both during acute psychosocial challenge tasks where observers’ emotional, autonomic and cortisol stress responses aligned with those of actively stressed individuals^5,10–12^, and in everyday life (in this context termed endocrine covariance; Engert et al.^13^). Vicarious stress, on the other hand, occurs when an observer experiences stress independently of the directly stressed individual’s experience, for instance, an observer’s stress levels can be high although the target directly experiencing the stressful situation exhibits low or no stress^5,11,12^. This vicarious stress response likely reflects the observer’s own stress sensitivity. In laboratory settings, acute stress resonance and vicarious stress are more likely to occur in couples (40%) compared to strangers (10%) and are stronger with direct rather than indirect exposure to the stressful situation^12^. Additionally, both stress resonance and vicarious stress have been positively associated with self-reported empathy^5,11,12^.

BBS is tightly linked to human social interactions. It can be observed across many different affiliative and cooperative contexts, where it is typically more pronounced in close relationships compared to interactions with strangers^9,14^. Traditionally, BBS is conceptualized as a reciprocal process emerging from bidirectional interactions between partners^9,15^. However, temporal alignment between physiological signals can also occur in less interactive contexts, for instance through emotional contagion, shared environmental cues, or attentional mechanisms^16^. Recent research has revealed preliminary evidence for a link between BBS and attachment representations^17,18^.

The identification of attachment types began within the domain of developmental psychology with studying children’s reactions to separation and reunion with their mothers in the “Strange Situation Procedure”^19^. These considerations were subsequently extended to adult attachment relationships, positing that Internal Working Models (IWMs) of attachment become more structured with age and are best understood through narrative assessment procedures^20^. Generally, three main organized attachment patterns are recognized: secure, insecure-preoccupied, and insecure-dismissing^20,21^. Secure attachment in adulthood is characterized by being comfortable in relationships and able to seek support if needed. Insecure-preoccupied attachment, in turn, is linked to excessive fear of abandonment and heightened support-seeking behaviors, whereas insecure-dismissing attachment is associated with emotional distance and a strong need for independence^20,21^. Beyond shaping relational patterns, attachment plays a crucial role as a resilience factor both individually and within a relationship, influencing how individuals cope with stress and adversity^6^. It not only shapes personal coping mechanisms in the face of stress and associates with lower acute stress sensitivity (e.g. Pierrehumbert et al.^22^), but also relates to empathic abilities (e.g. Stern and Cassidy^23^) which in turn facilitate support-giving within relationships^24^.

This dual role of attachment as a stress buffer and empathy booster makes it a compelling focus for investigation as both a resilience and potential risk factor in the context of empathic stress. While secure attachment is generally considered a resilience factor against stress, it may be less adaptive in scenarios of chronic relational stress—where higher empathic abilities may amplify the empathic stress load. In such cases, attachment security may predispose individuals to the excessive sharing of stress-related emotional and physiological activation, potentially transforming a protective factor into a risk factor.

Given the omnipresent nature of stress in daily life, this study aimed to elucidate the association between attachment and empathic stress sensitivity. We focused on acute stress in romantic couples within a psychosocial laboratory setting. Specifically, we investigated empathic stress—encompassing both stress resonance and vicarious stress—during a standardized paradigm where one partner underwent a psychosocial stress task (Trier Social Stress Test; Kirschbaum et al.^25^) while the other passively observed. By simultaneously capturing emotional, cardiovascular, and endocrine activation in both partners, we could directly link observers’ empathic stress responses to targets’ firsthand stress experiences. While synchrony is often conceptualized as a reciprocal process emerging from direct interaction, we consider a broader perspective that includes both reciprocal and unidirectional alignment. Specifically, we define synchrony here as the temporal relationship between two or more signals, without assuming causality or directionality, which allows us to capture co-regulation processes beyond direct interaction. In our study, this reflects the alignment between target and observer subjective stress responses, autonomous nervous system activity, and cortisol levels during simultaneous data collection, which may arise from interaction, shared environmental cues, or co-presence rather than direct bidirectional exchange^16^. Attachment was assessed with the Adult Attachment Interview (AAI; George et al.^26^), the current gold-standard narrative measure of adult attachment^27,28^. We hypothesized that inter-individual differences in attachment would be linked to empathic stress responses. Specifically, we proposed that observers with a secure (vs. insecure) attachment classification would exhibit lower levels of vicarious stress (a proxy of firsthand stress sensitivity), but that target-observer stress resonance would be higher in couples with securely (vs. insecurely) attached observers.

Given the established link of empathic abilities with attachment (e.g. Stern and Cassidy^23^) as well as with empathic stress^12^, we explored whether the hypothesized association between attachment and empathic stress would also be linked to higher levels of empathic abilities (particularly empathy, compassion, and Theory of Mind)^29^.

## Methods

### Participants

Eighty-five healthy, heterosexual couples participated in the study (*M*_age_ = 26.0, *SD* = 4.42 years, 50% women, sex self-reported). Study candidates underwent an initial telephone interview to screen for eligibility. We included individuals who Had been in a romantic relationship for at least 6 months, were between 20 and 40 years old, right-handed, and of normal weight (BMI < 30 and > 18). Individuals were excluded if they had previously participated in a standardized psychosocial stress task, were chronically ill (including current mental disorders), or taking steroid-containing or blood flow-changing medications. Additional exclusion criteria were regular cigarette smoking (> 5 cigarettes/week) and alcohol or recreational drug abuse. To screen for recreational drug use, participants underwent a rapid drug test upon arrival at the laboratory. Because the physiological stress response is also strongly influenced by sex hormones^30^, only women in the luteal phase of their menstrual cycle were tested. Women in any other cycle phase, taking hormonal contraceptives or pregnant were excluded. Except for left-handedness, all exclusion criteria were chosen as potential influencing factors of SAM or HPA axis reactivity or, in the case of mental and physical health, to assure that participants could handle the psychologically demanding stress task. Right-handedness was relevant for a functional near infrared spectroscopy (fNIRS) measurement that was taken but is not subject to the current manuscript. Inclusion criteria were consistent across all participants, irrespective of their roles in the experiment. All participants tested after October 2021 had to be vaccinated and present a negative COVID-19 antigen test result at study entry. The study was approved by the ethics committee of the Medical faculty of Leipzig University (EthicsID: 285/19-ek), and all methods were performed in accordance with the relevant guidelines and regulations, including the Declaration of Helsinki. Participants gave informed consent for their participation, could withdraw at any time, and were financially compensated. No data on ethnicity was collected.

Of the recruited N = 85 dyads, two AAIs could not be evaluated due to technical difficulties with the recording device; these participants also did not take part in the stress testing. Further, four participants dropped out for the stress testing because of changing life conditions such as break-up, moving to another city, and pregnancy. The sample of N = 79 dyads was further reduced to N = 66 for autonomous data due technical problems with the heart rate registration devices.

### Study design

Participants came to the laboratory on two testing days. Due to restrictions during the COVID-19 pandemic, the time gap between these two days varied considerably (*M* = 6.24 weeks; *SD* = 10.20 weeks). On one of the testing days, attachment and empathic processing were assessed with the Adult Attachment Interview (AAI; George et al.^26^) and the EmpaToM task^31^. Both targets and observers completed the 35-min EmpaToM task to assess different behavioral aspects of empathic processing (i.e., empathy, compassion, and Theory of Mind). Following the EmpaToM, only the observers underwent the AAI.

On the respective other testing day, participants underwent the empathic Trier Social Stress Test (E-TSST; Engert et al.^12^, Kirschbaum et al.^25^), a behavioral paradigm probing emotional and physiological stress resonance between two individuals (see section “Direct and empathic stress induction”). Since cortisol follows a circadian rhythm with highest levels immediately after awakening, testing was performed in the afternoon hours (between 12 and 6 pm). Next to emotional and peripheral markers of stress, simultaneous brain activation in targets and observers was recorded using fNIRS during the E-TSST; these data are not subject to the current manuscript and will be reported elsewhere.

The order of the two testing days was pseudo-randomized, such that 40 couples first attended the AAI and EmpaToM, and 39 couples first attended the empathic TSST. 6 couples only participated in AAI and EmpaToM. Likewise, observer sex was pseudo-randomized such that in 40 testing sessions observers were female.

### Direct and empathic stress induction

The Trier Social Stress Test (TSST; Kirschbaum et al.^25^) was used for acute stress induction. It is a standardized social-evaluative laboratory stressor in the form of a mock job-interview, and it reliably elicits subjective and physiological stress responses^32^. Compared to numerous alternative laboratory stressors, the TSST provokes the most robust HPA axis activation^33^. In detail, after a stress anticipation phase of variable length (5 min in our study), participants are instructed to give an audio- and video-taped 5 min free speech followed by a 5 min mental arithmetic task. Participants perform in front of a committee of two allegedly trained behavioral analysts, whom they believe evaluate their verbal expression, pitch, mimic, and posture, as well as the general quality of their performance.

**Fig. 1:**
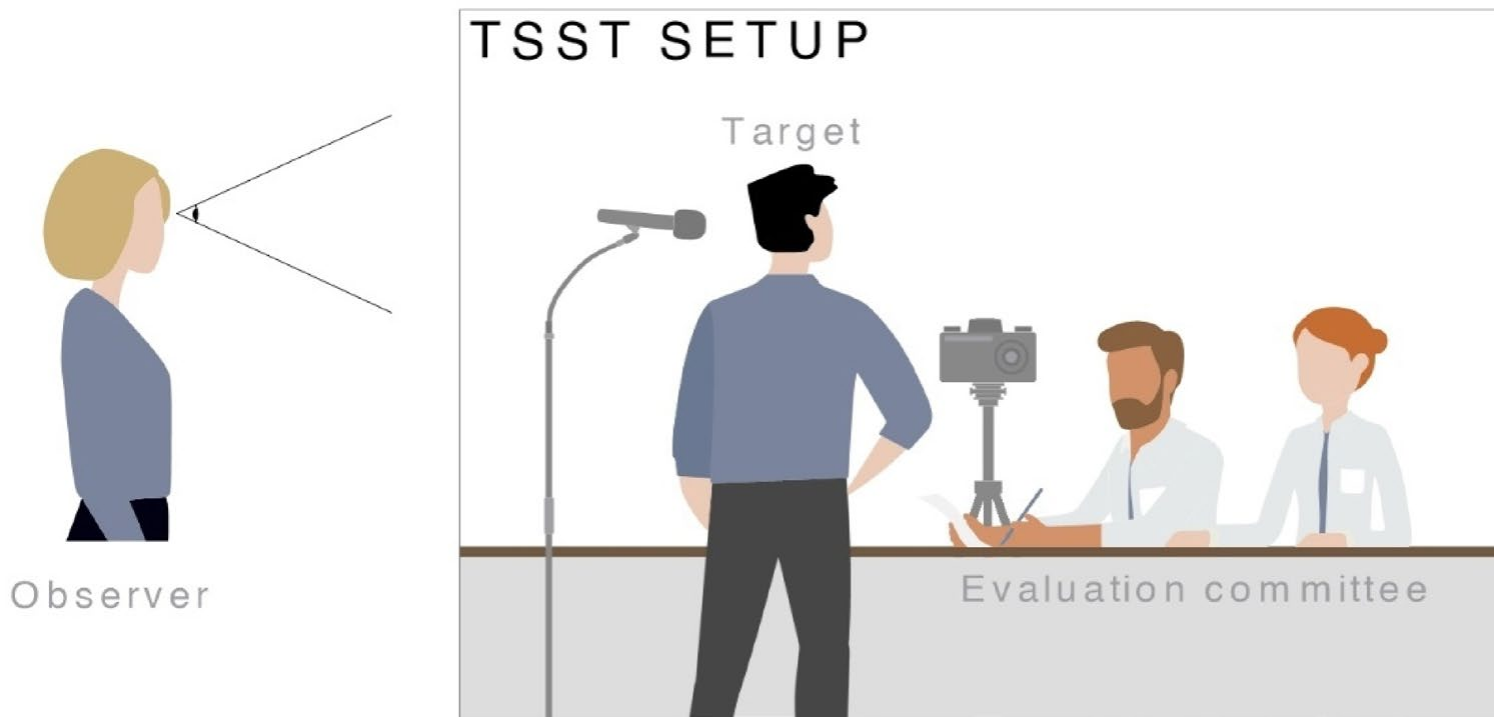
Empathic TSST Setup.

**Fig. 2:**
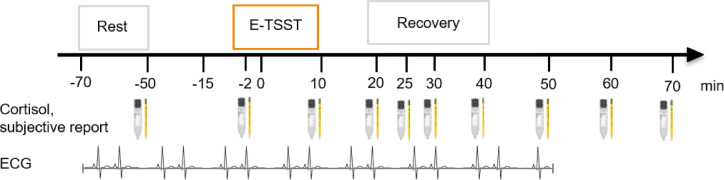
Stress testing timeline and assessed measures over time (relative to stressor onset at 0 min). The empathic TSST consisted of baseline, anticipatory, stress and recovery phases. 10 Subjective-psychological and cortisol measure were collected, cardiovascular measures were collected over a period of 115 min.

In the empathic version of the TSST (E-TSST; Engert et al.^12^) a passive onlooker observes the stressed target (Fig. 1). For the current study, participants were placed in two separate rooms during an initial 50-min resting phase prior to the actual testing period. This allowed them to recover from potentially stressful events experienced before entering the laboratory, and to desynchronize from one another (if they had arrived at the laboratory together). For the testing period, both participants were brought into the testing room where they were initially separated by a portable wall. After calibration of the fNIRS equipment, the portable wall was removed. The couple was then instructed to not interact with each other during the entire testing period. Observers were asked to passively observe the target. Targets were instructed to only look at the committee, not the observer. In this setup, the following three testing phases were executed: Baseline phase (non-stressful reading aloud task to set the fNIRS baseline; 5 min), stress anticipation phase (5 min), and stress phase, consisting of free speech and a mental arithmetic task (5 min each). Following the stress phase, observers completed a state empathy questionnaire. Both partners then separately left the testing room and rested alone during a 60-min recovery phase. At the end of the recovery phase, participants completed a set of online questionnaires. The detailed testing schedule is shown in Fig. 2.

Throughout the testing period, ten subjective emotional and ten cortisol samples were simultaneously collected in both partners. A continuous electrocardiogram (ECG) was recorded during 115 min from − 65 to 50 min (relative to stressor onset at 0 min). fNIRS measures and video recordings were taken during a 5-min baseline assessment, and then during the 5-min anticipatory and both 5-min stress phases.

Owing to hygienic restrictions on account of the COVID-19 pandemic, the TSST needed to be realized virtually for some dyads. This affected 15 out of the total number of 79 E-TSSTs conducted. In the virtual format, while both participants and TSST committee members came to the laboratory in person, the committee members (who were sitting together in a separate room) were projected at large scale into the testing room via video call. During the TSST instruction period, they shortly entered the TSST testing room to demonstrate the realness of the situation (and their actual presence) in the subsequent video-call.

### Adult Attachment Interview

Observers’ attachment representations were assessed with the Adult Attachment Interview (AAI; George et al.^26^), a 45–90 min semi-structured interview taking place in a one-on-one setting. It is considered to be the gold-standard of adult attachment measures and has excellent psychometric properties^27,28,34^. Focus of the AAI are early childhood experiences and the current evaluation and integration of these experiences into an individual’s personal biography. It encompasses the relationship to primary caregivers, experiences of separation and loss, and traumatizing events. Interviews were coded according to the AAI coding manual^35^ classifying attachment as secure or insecure (i.e., either insecure-dismissing or -preoccupied). Secure and insecure attachment representations are mainly distinguished by the ability to provide a comprehensive narrative without contradictions^35^. We used coherence of transcript (range 1 to 9) as an additional indicator of attachment security following a continuous approach (e.g., Bakermans-Kranenburg and van IJzendoorn^36^; Waters et al.^37^). Four trained interviewers conducted the interviews, which were audio recorded and transcribed verbatim. Transcripts were rated by two independent coders with reliability certification, being blind to all other data. Cross-reliability between the two independent coders was assessed in 15 interviews (18%). Coders achieved good agreement (κ = 0.61, *p* = .01) for AAI classification and the coherence of transcript scale (two-way mixed ICC = 0.88, *p* < .001).

### EmpaToM

The EmpaToM task^31^ effectively distinguishes three aspects of social processing: empathy, compassion, and Theory of Mind (ToM)^38^. During this 35-min task, participants view brief video clips where actors depict supposedly autobiographical events with variations in (a) emotional valence (negative or neutral) and (b) presence or absence of ToM demands. Each video lasts 15 s, with 12 trials per condition and 12 different narrators to mitigate narrator bias. After each video, participants rate their own affect (“How do you feel?”) and their level of compassion (“How much compassion do you feel?”). Following this, they answer questions about the video’s content: in the ToM condition, questions pertain to the narrator’s thoughts, goals, and intentions, whereas in the non-ToM condition, questions focus on factual details of the narrative. Participants also rate the confidence in their answers to measure meta-cognitive skills. Empathy was measured by assessing the increase in negative emotional responses after watching emotionally negative videos, calculated as the absolute difference between emotional ratings for negative and neutral videos. This approach allowed to account for participants’ overall mood. Compassion was evaluated by averaging the compassion ratings across all four video conditions. ToM ability was assessed through a combination of speed and accuracy: the z-scored means of ToM accuracy and reaction times were subtracted, and the result divided by two, thus controlling for individual response strategies (same procedure as used in Blasberg et al.^39^).

### Subjective stress experience

Subjective stress experience was assessed using the state form of the State Trait Anxiety Inventory (STAI; Spielberger et al.^40^), and a 7-point Likert scale asking for the level of currently experienced stress (“how stressed are you?”). The STAI is a 20 item-scale and the most frequently used instrument to measure stress-induced subjective-emotional states^41^. It asks for acute feelings of tension, worry, nervousness, and arousal. Participants filled out both scales at ten measurement time-points throughout the testing session. Relative to stressor onset (at 0 min) sampling took place at − 50 min (baseline), at − 2 min (immediately after stress anticipation and prior to the TSST), and at 10, 20, 25, 30, 40, 50, 60 and 70 min after stressor termination.

### Autonomic activity

Heart rate (HR) and high-frequency heart rate variability (HF-HRV) were assessed with a continuous electrocardiogram (ECG) using the Zephyr Bioharness 3 (Zephyr Technology, Annapolis, Maryland, USA), which is designed as a chest belt. It samples at a frequency of 250 Hz. Autonomic activity was recorded for 115 min in total, from − 65 to + 50 min relative to E-TSST onset (at 0 min). This time frame covered a 10-min baseline phase, a 5-min anticipation phase, the 10-min stress phase, and the 40-min stress recovery phase. The data were analyzed in corresponding 5-min intervals, with the results averaged across the entire duration of each interval. Data was corrected for artifacts using an in-house python script. The raw ECG data were manually reviewed by two independent research assistants for artifacts such as ectopic beats or signal loss. Segments of the ECG where heartbeats could not be reliably extracted were removed. If more than 10% of a specific phase had to be excluded, that phase was eliminated from further analysis. Subsequently, the average heart rate (beats per minute) and HF-HRV (in square milliseconds) were calculated for each 5-min interval. The python package “hrv-analysis” was applied to prepare ECG data and compute the average timeframes for heart rate and HF-HRV^42^.

### Salivary cortisol response

Cortisol is one of the primary biomarkers of the human stress response^1,43,44^. Cortisol levels were measured from saliva collected using Salivette collection devices (Sarstedt, Nümbrecht, Germany), which consist of a swab in a plastic container. At each measurement time-point, participants placed a collection swab in their mouth for 2 min and refrained from chewing. In order to ensure that samples were not contaminated with external particles, participants did not eat or drink anything other than water during the entire testing session. To equalize blood sugar levels, participants were given a snack upon arrival at the institute. Salivettes were stored at − 20 °C until analysis. Cortisol levels were assessed using a time-resolved fluorescence immunoassay with intra-/interassay variabilities of < 10%/12%^45^. Samples were analyzed at the Biochemical Laboratory of the Department of Biological and Clinical Psychology of Trier University.

### Statistical analysis

All analyses were performed with the software R, version 4.0.2^46^. Outliers were defined as values beyond 3 standard deviations from the mean. If not due to clear measurement errors, these values were winsorized to 3 standard deviations and included in the analysis. Cortisol and ECG data were log-transformed to normalize their distribution prior to winsorization. Data was checked for normality and equal variances.

For each stress marker (subjective-emotional stress, heart rate, HF-HRV, cortisol), reactivity change scores were calculated as the difference of average peak (i.e., value at time point at which most participants showed their highest value) minus baseline values. Given the strong influence of baseline levels on physiological reactivity (law of initial value; Wilder^47^), change scores were adjusted for levels at baseline by extracting the standardized change score residuals from a regression model that was fitted to the data of all participants (see approach in Engert et al.^12^). Reactivity scores thus captured peak stress while taking into account increases from baseline, making them a robust method to assess (empathic) stress responses in both target and observer. The use of stress reactivity scores as a primary measure of empathic stress is based on several previous empathic stress studies^10,12,48^. In our particular E-TSST setting, in which partners were only in the same room during the TSST anticipation and stress phases, reactivity scores were also suitable to capture maximal resonance, which should be relatively lower prior to the TSST and gradually decrease during the recovery period (both of which participants spent apart from each other). For autonomous markers, the significance level was Bonferroni-corrected to *p* = 0.025 since two variables were derived from one system and thus two comparisons were calculated. We did not correct for multiple testing via Bonferroni correction for the other markers because each stress marker is interpreted independently as a specific rather than a general case of empathic stress^49^. Analyses were preregistered at (aspredicted #120,537 https://aspredicted.org/zkqy-2wb6.pdf ). Originally, we preregistered the study for 80 dyads. However, to compensate for partial drop-outs in the context of the TSST, we increased the study sample to N = 85 dyads, resulting in N = 79 dyads available for the empathic stress analyses.

#### Sensitivity Analyses

To assess whether our sample size was adequate for detecting small to medium effects, we conducted a sensitivity analysis with G*Power^50^. With a total of 79 dyads, a power level of 0.80, and an alpha of 0.05, the analysis showed that medium to large effects (f ≥ 0.30) could be reliably detected which were the effect sizes expected for our analyses^12^. However, to detect smaller effects (f = 0.10), a larger sample size of 202 dyads would have been necessary.

#### Attachment classification

To examine the presence of empathic stress and the association between attachment and empathic stress, a linear model was calculated for each stress marker (4 in total; for subjective-emotional stress, heart rate, HF-HRV, and cortisol) using the average observer reactivity change scores as dependent variables and the target reactivity change scores as predictors. Attachment classification derived from the AAI was then entered into each model as a categorical predictor (secure, insecure), and as an interaction with the target reactivity change score of the corresponding stress marker. In these models, a main effect of attachment was interpreted as an influence of attachment on the occurrence of vicarious stress (observer stress reactivity independent of target stress reactivity). An interaction of attachment with the target reactivity change score was interpreted as an influence of attachment on the occurrence of stress resonance (observer stress reactivity depending on target stress reactivity). Control variables with a known influence on the respective variables of interest were added to all models. For cortisol, these included sex^51^ and time of day^52^, for autonomic markers, sex^53^ and body mass index (BMI; Molfino et al.^54^). In addition to the preregistered winsorization of outliers, we assessed the robustness of our linear models by visually inspecting extreme data points. In the HF-HRV analysis, we identified a change score outlier that exerted disproportionate influence on the model estimates. To ensure that our findings were not unduly driven by individual extreme values, we examined the effect of removing this outlier, which rendered a previously significant effect non-significant. Although only this outlier affected significance, we removed a second extreme value for consistency in data treatment (Supplementary Fig. S1).

*Exploratory association with Empathic Abilities.* To elucidate the role of empathy, associations of attachment and stress resonance were followed up with exploratory analysis in three linear models including interaction terms of target stress reactivity, attachment, and empathic abilities derived from the EmpaToM: empathy (1), compassion (2), and Theory of Mind (3).

#### Additional analyses

*Intradyad correlations.* There is currently no best practice in how to analyze dyadic empathic stress data. In the preregistration of this paper (aspredicted #120,537), we had suggested the use of intradyad correlations as an additional method to gauge the amount of resonance between targets and observers. Intradyad correlations for subjective stress, heart rate (HR), high-frequency heart rate variability (HF-HRV), and cortisol were calculated using the Pearson correlation coefficient, based on all available measurement time points for each couple. These correlations reflect the degree of synchronized variation between partners’ stress responses, rather than the intensity of any individual participant’s stress response (procedure as in Engert et al.^13^). We replicate the above main models using intradyad correlations as dependent variable in the Supplemental Material.

*Coherence of transcript.* In an exploratory approach to verify the consistency of the above results, all described analyses were additionally calculated with the continuous attachment variable coherence of transcript (replacing the categorical attachment classification from the AAI).

## Results

### Preliminary analyses

#### Descriptive statistics

The N = 79 tested dyads (158 participants) Had a mean age of 26.11 ± 4.40 years. Among the observers, 62% were securely and 38% insecurely attached. The majority of the insecurely attached observers were classified as insecure-dismissing; only two observers (2%) were classified as insecure-preoccupied and one received an additional unresolved label. Sensitivity analysis revealed consistent results when excluding the individuals with insecure-preoccupied and unresolved categorization. The two main attachment groups statistically significantly differed in sex, with more men categorized as insecurely attached (t-test; *t*(75) = 2.78, *p* = .007), but not in age (t-test;* t*(75) = 0.48, *p* = .63). In the reduced sample for autonomous data comprised of N = 64 dyads, mean age was 26.05 ± 4.43 years. 64% observers were classified as securely attached, 36% as insecure, out of which the majority was insecure-dismissingly attached. The two participants classified as insecure-preoccupied, and the one classified as insecure-dismissing with an additional unresolved label remained in the sample. Again, the two main attachment groups statistically significantly differed in sex, with more men categorized as insecurely attached (t-test; *t*(62) = − 2.63, *p* = .01), but not in age (t-test;* t*(62) = 0.11, *p* = .91).

#### Verification of successful (empathic) stress induction

Among the targets, 77% showed a physiologically significant cortisol stress response of at least 1.5 nmol/l over baseline levels^55^, indicating successful stress induction. The TSST responder rate was consistent with the expected range of 70–85% (^32^). Among the observers, 24% (19 participants) were significant cortisol responders. In the virtual TSST, the responder rate among targets was 87%, and 27% among observers. In the non-virtual setting, the responder rate for targets was 75%, and 23% for observers. These percentages of observers showing physiologically significant cortisol stress responses was lower than in our earlier empathic stress study (40% in partner dyads^12)^. Since we expected empathic stress responses to vary in intensity, and synchrony to occur in dyads with minimal cortisol release, all participants were included in subsequent analyses, even if they did not reach the threshold of a full-blown stress response.

To verify the occurrence of stress resonance, we calculated linear models for all stress markers with observer reactivity as dependent and target reactivity as independent variables. For cortisol, we found that target reactivity significantly predicted observer reactivity (*t*(75) = 2.49, *p* = .015; Table 1). We could thus confirm the occurrence of cortisol stress resonance in our sample.

**Table 1:** Linear models for observers’ stress reactivity (change score) as dependent variable; targets’ stress reactivity (change score) as predictor, for acute subjective stress, autonomous markers heart rate (HR) and high-frequency heart rate variability (HF-HRV), and salivary cortisol.

	Subjective Stress	HR	HF-HRV	Cortisol
Intercept	− 0.02	1.58	1.20	0.45
Target change score	− 0.09	− 0.15	− 0.07	0.28*
BMI	−	− 0.07	− 0.05	−
Sex	–	− 0.21	0.27	− 0.13
Time of day	–	–	–	< 0.01
R^2^	0.01	0.04	0.03	0.11

***p ≤ .001, **p ≤ .01, *p ≤ .05; for HR and HF-HRV after Bonferroni correction: ***p ≤ .0005, **p ≤ .005, *p ≤ .025, *p* ≤ .05..

### Empathic stress and attachment

#### Attachment classification

**Fig. 3:**
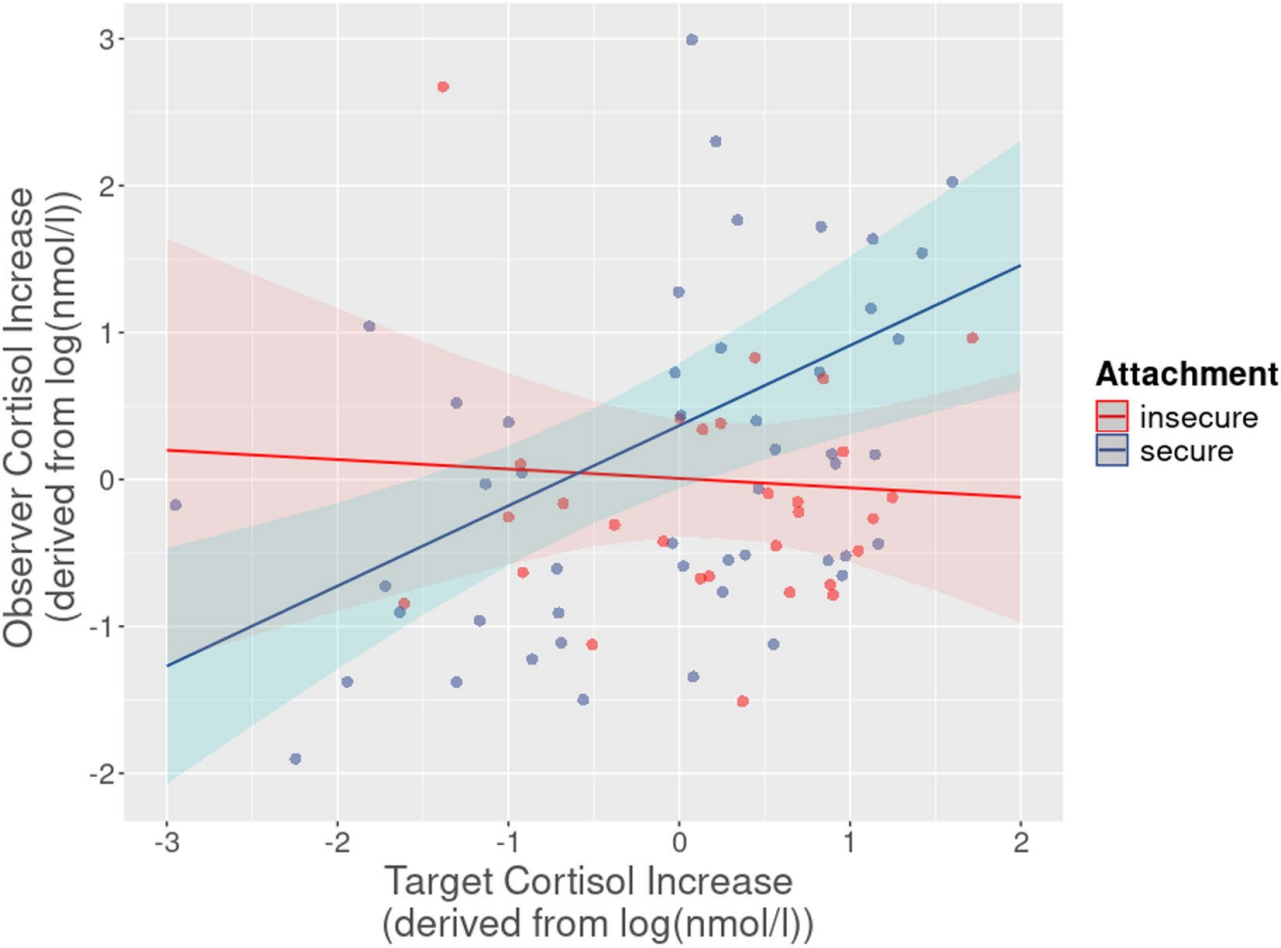
Association of stress resonance with attachment. Linear models revealed that secure observer attachment (colored lines) correlated with higher dyadic stress resonance in acute cortisol reactivity (*t* = 2.27, *p* = .026). Stress reactivity was calculated as residualized change score peak minus baseline. Observer attachment type was assessed with the Adult Attachment Interview (AAI) categorically (secure/insecure)^26^. Shading = 95% confidence intervals.

To investigate the association between attachment and empathic stress, we first calculated linear models with observer stress reactivity as the dependent variable, and observer attachment representation (secure vs. insecure) and target stress reactivity (main effect and interaction with observer attachment) as predictors. We found a significant interaction between observer attachment and target stress in cortisol (*t* = 2.27, *p* = .026; Fig. 3), indicating higher cortisol stress resonance in dyads with securely attached observers. Tukey posthoc tests revealed significant target-observer associations specifically in dyads with securely attached observers for cortisol (secure attachment: *r* = 0.43, *p* = .002; insecure attachment: *r* = − 0.06, *p* = .75). No statistically significant effects were found in subjective stress, heart rate, or HF-HRV (Table 2).

**Table 2:** Linear models for observers’ stress reactivity (change score) as dependent variable; targets’ change score, categorial observer attachment classification (secure/insecure) and their interaction as predictors, for acute subjective stress, autonomous markers heart rate (HR) and high-frequency heart rate variability (HF-HRV), and salivary cortisol.

	Subjective Stress	HR	HF-HRV	Cortisol
Intercept	0.07	1.02	0.60	0.13
Target change score	− 0.11	0.43	0.31	− 0.06
Observer attachment (categorial: secure/insecure)	− 0.23	− 0.02	− 0.03	0.36
Target change: AAI attachment (categorial: secure/insecure)	0.05	− 0.46	− 0.30	0.61*
BMI	**–**	− 0.04	− 0.03	–
Sex	–	− 0.24	0.09	− 0.43
Time of day	–	–		< 0.01
R^2^	0.02	0.06	0.07	0.13

***p ≤ .001, **p ≤ .01, *p ≤ .05; for HR and HF-HRV after Bonferroni correction: ***p ≤ .0005, **p ≤ .005, *p ≤ .025, *p* ≤ .05..

#### Exploratory association with empathic abilities

Due to there being resonance for cortisol, we calculated three further linear models in which we implemented a triple interaction of target cortisol change score, observer attachment, and empathic abilities: empathy (1), compassion (2), and Theory of Mind abilities (3), to investigate the role of empathic abilities in the link between attachment and stress resonance. Observer stress reactivity change scores were again entered as dependent variable into these models. No statistically significant triple interactions were found (Table 3).

**Table 3:** Three linear models for observers’ stress reactivity (change score) as dependent variable; targets’ stress reactivity (change score), observer attachment classification (secure/insecure), empathic abilities (Empathy (1), Compassion (2), ToM: theory of mind abilities (3)), and their interaction as predictors, for salivary cortisol.

	Empathy (1)	Compassion (2)	ToM (3)
Intercept	0.08	− 1.82	0.17
Target change score	0.34	2.43*	− 0.16
Observer attachment	0.79	2.13	0.40
Empathic ability_Empathy (1), Compassion (2), or ToM (3)_	0.01	0.03	0.40
Target change: Observer attachment	0.30	− 1.37	0.70*
Target change: Empathic ability_Empathy (1), Compassion (2), or ToM (3)_	− 0.02	− 0.03*	− 3.36
Observer attachment : Empathic ability_Empathy (1), Compassion (2), or ToM (3)_	− 0.02	− 0.28	− 0.48
Target change: Observer attachment: Empathic ability_Empathy (1), Compassion (2), or ToM (3)_	0.02	0.03	3.89
Sex	− 0.42	− 0.41	− 0.45
Time of day	< 0.01	< 0.01	< 0.01
R^2^	0.22	0.28	0.23

***p ≤ .001, **p ≤ .01, *p ≤ .05..

#### Additional analyses

*Intradyad correlations.* There were no statistically significant effects of intradyad correlations in our stress markers with observer attachment security (categorial) (Table S1).

*Continuous attachment variable coherence of transcript.* Aiming to verify the stability of our main results, the above-described models were repeated using the continuous AAI observer attachment variable coherence of transcript. Mirroring the results of the main model for cortisol, we found an interaction effect between coherence of transcript and the target stress reactivity score, indicating higher stress resonance in dyads with observers scoring higher on attachment security (Table S2). Additionally, in the intradayd correlations, a significant effect of coherence of transcript on intradyad correlations for cortisol emerged, suggesting higher observer attachment security to be associated with higher intradyad correlations (Table S3), and again confirming observer attachment security to be associated with higher cortisol resonance.

## Discussion

In the current study, we used a multimodal approach to investigate the association between attachment representations and empathic stress in a situation of acute psychosocial stress in romantic couples. Deepening our understanding of these dynamics may be an important steppingstone in boosting resilience in romantic relationships, thus enabling couples to manage and recover from (empathic) stress more effectively.

Our findings showed resonance in cortisol stress responses, indicating the proportional release in cortisol in passive observers and directly stressed targets. Also, observer attachment was shown to significantly associate with stress-induced biobehavioral synchrony (BBS) in cortisol. Particularly, secure (vs. insecure) observer attachment was associated with higher dyadic cortisol stress resonance. No resonance (or association with attachment) was found for emotional and autonomous stress reactivity. Also, observers’ vicarious stress (a proxy of firsthand stress sensitivity, independent of target stress reactivity) was unrelated to their attachment representations.

Our finding of higher stress resonance in dyads with observers classified as securely attached is in line with our a priori hypothesis, which was based on attachment theory. Securely attached individuals typically hold positive internal working models (IWMs) of attachment of the self and others. They expect others to possess good intentions, trust them, and view themselves as competent in both seeking for and providing support under distress^56^. This positive belief system encourages care for others and prevents hostile behavior^23^. In our study, the positive belief system may be reflected in relatively higher physiological attunement with the romantic partner’s acute stress. Conversely, insecure-dismissingly attached individuals tend to have negative perceptions of others^57^, exhibit reduced emotional understanding^29^, and, in their aim to prioritize their own independence, may disregard others’ needs and emotions^58^. These tendencies are reflective of a deactivation of the attachment system not only to minimize emotional responsiveness and the emerging need to seek others’ support under distress^59^, but also with deactivating and distancing caregiving^60^. Such deactivation may contribute to the lack of stress resonance seen in insecure-dismissingly attached partners.

A potential contributing factor to the attachment-related differences in stress resonance could be attachment-related differences in empathic abilities. We know from prior research that empathic abilities are crucial in modulating BBS in general^61^, and more specifically in the context of stress resonance^10–12^. Moreover, in their meta-analysis, Xu et al.^62^ revealed a positive interaction between empathy and secure attachment, and a negative interaction between empathy and insecure-dismissing attachment. In a prior study in the same sample as tested here, we furthermore discovered that insecure-dismissing attachment, as measured with the Adult Attachment Interview (AAI), was linked to diminished cognitive empathy, specifically Theory of Mind abilities assessed in the EmpaToM^29,31^. However, empathic abilities did not interact with the link of attachment and stress resonance found in the current data. There are several possible explanations for this null result. First, previous findings of an association between the occurrence of empathic stress and empathic abilities were questionnaire-based rather than behavioral (i.e., EmpaToM-based). Touching upon the same issue as found in the attachment field (i.e., the lack of a correlation between self-report questionnaires and narrative, arguably more objective measurement methods, Roisman et al.^63^), questionnaire-based and behavioral empathy data do not reliably correlate^64^. Further, attachment could influence both empathic abilities and stress resonance independently, without a mediating role.

Emotional closeness is another potential contributing factor to the link between attachment and the tendency to exhibit stress resonance. Thus, when comparing romantic partners and strangers in our earlier study, emotional closeness was a modulator in the occurrence of stress resonance^12^. Moreover, individuals with insecure-dismissing attachment have been found to perceive others as less emotionally close compared to securely attached individuals^65^. Because we focused on romantic partners in the current study and did not assess emotional closeness, we cannot further specify a potential role of emotional closeness.

The emotional context seems to be crucial for the occurrence of BBS, including physiological synchrony. In our study, the utilized external stressor only directly affected one relationship-partner. Our experimental setup therefore differed from other studies that used more directly relationship-related stressors affecting both partners and found that higher cortisol synchrony was linked to lower relationship satisfaction^66^, lower levels of support behavior^67^, and was predictive of breakup^68^. When considering the resilience-promoting potential of attachment security, lower cortisol attunement in a relationship conflict affecting both partners may indicate that one partner is effectively managing the conflict by remaining calm and composed, thereby providing a de-stressing, stabilizing influence on the couple. Higher cortisol attunement when confronted with an external stressor that only directly affects one partner, on the other hand, may have somewhat different adaptive effects. For example, it may allow for the mobilization of the necessary energetic resources in the observer to overcome adversity together with the target after the stressor has been removed^69^. Resonance in a situation of external stress influence may also foster a better understanding of the partner’s emotional state. By showing similar emotional and bodily experiences, individuals may better empathize and connect with the other, enhancing interpersonal relationships and promoting mutual support^5^. Further, stress resonance may serve as a warning signal in situations of danger. By perceiving and resonating with the stress experienced by others, individuals may detect potential risks and adjust their own behavior accordingly, promoting safety and self-preservation^70^. Another factor to consider within the emotional context of the E-TSST is that the stress resonance observed in securely attached individuals may arise from the distress of being unable to help their partner in distress.

Lastly, our models indicated that securely attached individuals exhibited cortisol resonance also in the absence of stress in both partners (i.e., low target stress correlated with low observer stress), indicating a heightened synchrony during non-stressful periods. These data could suggest that securely attached individuals may regulate homeostatic processes in synchrony, encompassing both stressful and non-stressful phases. This propensity could serve as a protective mechanism, mitigating the long-term stress burden.

Despite its many protective properties, attachment security may bear potential risks when it comes to a context of increased suffering or stress exposure in the context of empathic stress. The permanent confrontation with the stress of others, conceivable in the partners or children of chronically stressed individuals, or in people working in care professions, may lead to an increased physiological stress load in the empathic observer. Given the critical role of heightened cortisol levels in the development of stress-associated disease^1–3^, long-term stress resonance may put individuals at risk for adverse health outcomes themselves. Thus, while covariation in which couples influence each other toward homeostasis may promote health and well-being^71^, physiological linkage with chronically stressed loved ones may predispose affected individuals to HPA-axis dysregulation themselves. In this context, the emotional toll of caregiving can extend beyond empathic stress to outcomes like secondary trauma and compassion fatigue, although this term may be misleading. Research increasingly suggests that compassion itself is associated with positive health outcomes and does not necessarily lead to fatigue^72^. Secondary trauma arises when caregivers internalize the distress of others^73^, while compassion fatigue reflects emotional exhaustion from prolonged caregiving^74^. Both are linked to sustained exposure to others’ suffering and, like empathic stress, can pose serious health risks if unaddressed. To prevent these outcomes, interventions such as compassion training offer promise. Unlike empathy, which can lead to emotional overload, compassion fosters both care and resilience, allowing individuals to engage in caregiving without succumbing to burnout^75^. Emerging evidence suggests that developing compassion can help protect caregivers from the detrimental effects of secondary trauma and compassion fatigue^76^.

Other than expected, we found no association of observer vicarious stress (i.e., observer firsthand stress sensitivity, independent of target stress reactivity) with observer attachment. Our a priori hypothesis of higher vicarious stress in insecurely attached individuals had been based on previous research demonstrating higher cortisol reactivity to direct stress exposure in individuals with an insecure-preoccupied attachment^38,77–80^. Since insecure-preoccupied individuals were clearly underrepresented in our sample, there was likely insufficient power to detect the expected effect. In contrast to insecure-preoccupied individuals who are prone to the use of hyperactivating regulatory attachment strategies, insecure-dismissing individuals tend to distance themselves from others and rely more on independence, employing deactivating attachment strategies such as suppressing their emotional needs and minimizing the importance of close relationships^59^. All of these behaviors may render them less rather than more vulnerable to the experience of vicarious stress.

The specificity of the current resonance finding for cortisol reactivity suggests that due to its slower response dynamics (compared to autonomic nervous system or emotional reactivity), cortisol may be particularly well-suited to capturing empathic stress responses. Unlike autonomic and emotional reactions, which fluctuate within seconds to minutes, cortisol peaks 20–40 min post-stressor, reflecting broader regulatory processes rather than immediate emotional attunement. While this aligns with previous research in adult dyads^12^, recent studies in mother–child and parent-adolescent dyads contrarily showed resonance only in terms of parasympathetic reactivity^10,81^. Additional research is needed to understand whether adults and children show systematically different patterns of empathic stress responding depending either on endocrine or autonomic resonance. An alternative explanation is that cortisol synchrony may not solely reflect stressor-specific resonance but rather general dyadic synchrony, as suggested by research on couples’ physiological attunement^82^. In this view, habitual co-regulation or shared environmental influences may drive cortisol alignment, even in the absence of a subjective stress response.

A limitation of our study is the lack of a large enough insecure-preoccupied participant group. Future studies should expand their scope to include individuals with insecure-preoccupied attachment, as these exhibit unique internal working models and behaviors. Further, measuring emotional closeness, intimacy and relationship difficulties would have been beneficial to further understand the found associations. Additionally, investigating target attachment to assess its impact on target stress, as well as exploring the interactions between target and observer attachment on the observer effect, would have provided valuable insights into relationship dynamics in the context of empathic stress. However, executing Adult Attachment Interviews also in the targets of the current study was not feasible due to limited resources. Also, our study design did not allow for a direct assessment of bidirectional biobehavioral synchrony, as observers were instructed to focus on the committee rather than directly engaging with the targets. While our approach captures temporal alignment between physiological signals, it does not fully reflect interactive synchrony as traditionally defined. Future studies should implement paradigms that enable direct social interaction to further examine reciprocal regulatory processes. Lastly, our study was limited by a lack of diversity in the sample, as we only included healthy, heterosexual couples.

In sum, we provide evidence for a link of attachment with the occurrence of empathic stress, such that dyads of romantic partners with securely (as opposed to insecure-dismissingly) attached observers exhibited higher resonance during acute psychosocial stress experience. This association between observer attachment and empathic stress was specific to cortisol responding, and hence not observed in subjective-psychological or autonomic stress reactivity. Observer attachment was also unrelated to the strength of observer vicarious stress responding. Higher cortisol resonance may have multiple adaptive functions, such as fostering mutual understanding and facilitating partner support. Conversely, it may have negative health implications in situations of chronic exposure to others’ stress. Further research is needed to explore the long-term consequences of attachment-related empathic stress on health and well-being, and to investigate the underlying mechanisms driving these effects. By elucidating the role of attachment in empathic stress, we may contribute to the development of targeted interventions aimed at promoting both stress resilience and healthier relationships.

## Supplementary Information

Supplementary Information.

## Data Availability

The data used in this study, including attachment, empathic abilities, physiological measures, and questionnaire responses, are available at the following link: https://osf.io/faeh4/?view_only=754b8b17acbf4d889e205b29f44bdaf7.
