# Supplementary material for: Securely stressed: association between attachment and empathic stress in romantic couples
Source: Sci Rep. 2025 Sep 12;15:32420. doi: 10.1038/s41598-025-13970-9 (PMC12432227; doi:10.1038/s41598-025-13970-9)
Supplement: Supplementary file 1 — Supplementary Information. [file 41598_2025_13970_MOESM1_ESM.docx]

**Supplementary Material for**

Securely Stressed: Association between Attachment and Empathic Stress in Romantic Couples

**Figure S1. Outlier Removal Process in HF-HRV Analysis.** (A) Linear model including identified outliers. (B) Linear model after removing an outlier that rendered the result non-significant. (C) Second outlier removed for consistency, without affecting significance. The three figures show the association between HF-HRV changes of target and observer with observer attachment, modeled using residualized change scores (peak minus baseline). Observer attachment (colored lines) was assessed categorically (secure/insecure) using the Adult Attachment Interview (AAI; George et al., 1985). Shaded areas represent 95% confidence intervals.


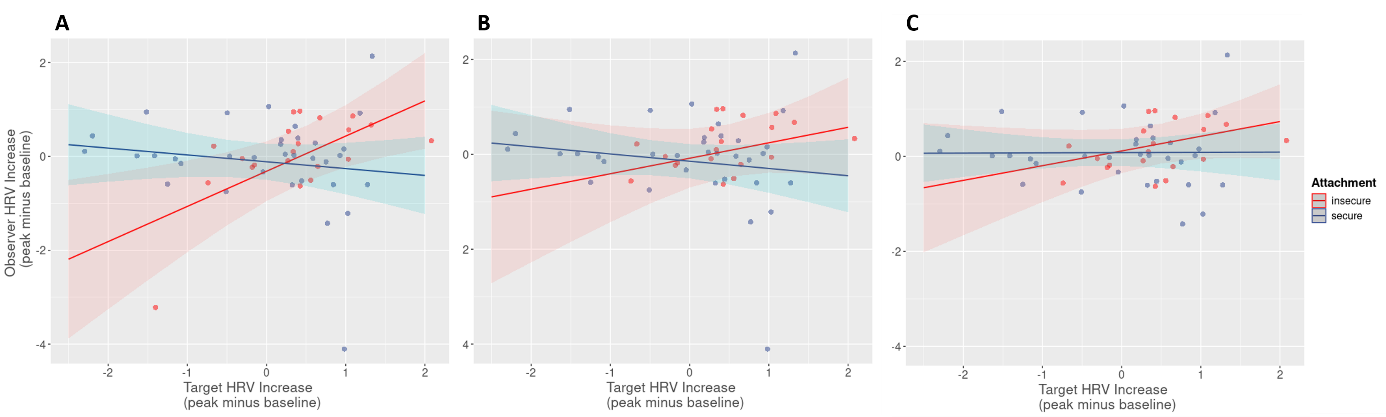


**Table S1.** Linear Models for dyadic correlations with attachment classification (secure/insecure) as predictor for acute subjective stress, autonomous markers heart rate (HR) and high-frequency heart rate variability (HF-HRV), and salivary cortisol.

|  | Subjective Stress | HR | HF-HRV | Cortisol |
| --- | --- | --- | --- | --- |
| Intercept | 0.54 | 0.49 | 0.71 | 0.34 . |
| Observer Attachment (categorial: secure/insecure) | -0.11 | -0.02 | -0.08 | -0.17 |
| BMI | - | -<0.01 | -0.01 | - |
| Sex | - | <0.01 | -0.15 | -0.07 |
| Time of day | - | - | - | -0.0001 |
| R^2^ | 0.02 | 0.004 | 0.02 | 0.03 |

*Note: ***p ≤.001, **p≤.01, *p≤.05;
 for HR and HF-HRV after Bonferroni correction: ***p≤.0005, **p≤.005, *p≤.025, . p≤.05*

**Table S2.** Linear Models for observers’ change score as dependent variable; targets’ change score, continuous attachment variable „coherence of transcript“ and their interaction as predictors, for acute subjective stress, autonomous markers heart rate (HR) and high-frequency heart rate variability (HF-HRV), and salivary cortisol.

|  | Subjective Stress | HR | HF-HRV | Cortisol |
| --- | --- | --- | --- | --- |
| Intercept | -0.26 | 1.77 | 0.56 | -0.15 |
| Target Change Score | -0.40 | 0.20 | 0.63 | -0.71. |
| Observer Attachment  (continuous: coherence of transcript) | 0.05 | -0.03 | 0.01 | 0.12. |
| Target Change: Observer Attachment (continuous) | 0.06 | -0.06 | -0.10 | 0.22** |
| BMI | - | -0.07 | -0.03 | - |
| Sex | - | -0.14 | 0.09 | -0.45. |
| Time of day | - | - | - | -<0.01 |
| R^2^ | 0.03 | 0.04 | 0.08 | 0.23 |

Note: ***p ≤.001, **p≤.01, *p≤.05;
 for HR and HF-HRV after Bonferroni correction: ***p≤.0005, **p≤.005, *p≤.025, . p≤.05

**Table S3.** Linear Models for dyadic correlations with continuos attachment variable „coherence of transcript“ as predictor for acute subjective stress, autonomous markers heart rate (HR) and high-frequency heart rate variability (HF-HRV), and salivary cortisol.

|  | Subjective Stress | HR | HF-HRV | Cortisol |
| --- | --- | --- | --- | --- |
| Intercept | 0.35 | 0.64 | 0.48 | -0.15 |
| Observer Attachment (continuous: coherence of transcript) | 0.03 | -<0.01 | 0.02 | 0.09* |
| BMI | - | -0.01 | -0.01 | - |
| Sex | - | -0.05 | -0.15 | 0.11 |
| Time of day | - | - | - | <0.01 |
| R^2^ | 0.02 |  |  | 0.08 |

Note: ***p ≤.001, **p≤.01, *p≤.05;
 for HR and HF-HRV after Bonferroni correction: ***p≤.0005, **p≤.005, *p≤.025, . p≤.05

**References**

George, C., Main, M., & Kaplan, N. (1985). Adult attachment interview (AAI)[Database record]. *University of California, Berkeley: APA PsycTests*.
